# A Practical Treatise on Diseases of Women

**Published:** 1874-12

**Authors:** 


### Books Reviewed.

*A Practical Treatise on Diseases of Women.* By T. Gaillard Thomas, M. D., etc. Fourth Edition Thoroughly Revised, with one hundred and ninety-one illustrations on wood. Philadelphia: Henry C. Lea, 1874. Buffalo: T. Butler & Son.

Dr. Thomas' excellent work has passed through three large editions in this country, and has been translated into German; preparations are also being made to render it in French and Italian. These facts, better than anything which we can say, show the high appreciation which professional readers have of the work.

The present edition is a thorough revise of the last one, many changes and additions being made. The illustrations have been in many instances improved over those of the former edition, and many have been entirely omitted.

In his preface the author urges upon the profession, the lateral position and Sims' speculum, for all vaginal examinations and manipulations.

This position is the one to which the author refers thoughout the work, and the one which he employs in private and hospital practice.

In the portion of the work devoted to uterine displacements, the author seems to favor the use of pessaries and various uterine supporters, a practice which with some physicians is becoming too much a fashion; in Professor Thomas' hands it may be safe practice, in some it is far from it.

The author gives Prof. J. P. White, of this city, tardy credit for his valuable and very successful mode of reducing inversion of the uterus, and on page 439 figures a uterine repositor suggested by Prof. White. He seems however ignorant of the cases reported by Dr. W. at the meeting of the New York State Medical Society, one of which was of twenty-two years duration.

He speaks highly, as he did in the last edition, of the method of ovariotamy by enucleation, but seems to be laboring under a misapprehension as to its adaptability to all cases, and the mode of operating. On page 752, he seem to limit its adaptability to cases having no pedicle, this probably is not the authors true meaning as on pages 743, 744, he advises its use where the pedicle is short and very vasular. The advice to touch all the bleeding points after enucleation seems unnecessary as when slowly and carefully performed, no hemorrhage occurs than cannot be checked by more simple means.

The entire work is an important and valuable addition to the literature of gynæcology, and is so well and favorably known through former editions that it needs no praise from us. The American profession may however feel proud that such a work emenates from one of its members.

